# Homocysteine, Grey Matter and Cognitive Function in Adults with Cardiovascular Disease

**DOI:** 10.1371/journal.pone.0033345

**Published:** 2012-03-07

**Authors:** Andrew H. Ford, Griselda J. Garrido, Christopher Beer, Nicola T. Lautenschlager, Leonard Arnolda, Leon Flicker, Osvaldo P. Almeida

**Affiliations:** 1 Western Australian Centre for Health & Ageing, Centre for Medical Research, University of Western Australia, Perth, Western Australia, Australia; 2 School of Psychiatry and Clinical Neurosciences, University of Western Australia, Perth, Western Australia, Australia; 3 Department of Psychiatry, Royal Perth Hospital, Perth, Western Australia, Australia; 4 School of Medicine and Pharmacology, University of Western Australia, Perth, Western Australia, Australia; 5 Academic Unit for Psychiatry of Old Age, St. Vincent's Health, Department of Psychiatry, St. George's Hospital, The University of Melbourne, Melbourne, Victoria, Australia; 6 Academic Unit of Internal Medicine, Australian National University Medical School, Canberra, Australian Capital Territory, Australia; Federal University of Rio de Janeiro, Brazil

## Abstract

**Background:**

Elevated total plasma homocysteine (tHcy) has been associated with cognitive impairment, vascular disease and brain atrophy.

**Methods:**

We investigated 150 volunteers to determine if the association between high tHcy and cerebral grey matter volume and cognitive function is independent of cardiovascular disease.

**Results:**

Participants with high tHcy (≥15 µmol/L) showed a widespread relative loss of grey matter compared with people with normal tHcy, although differences between the groups were minimal once the analyses were adjusted for age, gender, diabetes, hypertension, smoking and prevalent cardiovascular disease. Individuals with high tHcy had worse cognitive scores across a range of domains and less total grey matter volume, although these differences were not significant in the adjusted models.

**Conclusions:**

Our results suggest that the association between high tHcy and loss of cerebral grey matter volume and decline in cognitive function is largely explained by increasing age and cardiovascular diseases and indicate that the relationship is not causal.

## Introduction

Homocysteine is an amino acid derived from dietary-obtained methionine through demethylation [1]. Elevated total plasma homocysteine (tHcy) has been implicated in a number of disease processes including vascular diseases [2], dementia [3], cognitive impairment [4], Raynaud's phenomenon [5], osteoporotic fractures [6], heart failure [7] and depression [8].

The observation that elevated tHcy is associated with vascular disease dates back to the 1960s [9]. High tHcy increases the risk of cerebrovascular disease, stroke and ischemic heart disease [10], thromboembolic disease [11] and peripheral vascular disease [12]. It has also been associated with higher risk of congestive heart failure independent of the presence of concurrent ischemic heart disease [13]. In addition, a wealth of observational data indicates an association between elevated tHcy and cognitive impairment [14], although the few randomized controlled trials available to date of homocysteine lowering treatment with B-vitamins have generally failed to reduce the rate of cognitive decline in people at risk [15].

Elevated tHcy has also been associated with anatomical and neuroimaging changes of the brain. Data from the Sydney Stroke Study showed that people with high tHcy have larger anterior ventricle-brain ratio after controlling for age, folate, vitamin B12, creatinine and white matter disease (OR = 2.3, 95%CI 1.03–5.09) [16]. High tHcy has also been associated with silent brain infarcts and white matter lesions [17], and vascular disease is a well-established risk factor for cognitive impairment and dementia [18].

There are numerous hypotheses as to how elevated tHcy promotes brain atrophy and increases the risk of cognitive impairment and dementia, independent of its role in the pathogenesis of vascular disease [19]. However, the effect of elevated tHcy on brain structure and function once prevalent cardiovascular diseases are taken into account remains uncertain.

We designed this study to determine if high tHcy is associated with lower cognitive function and with loss of cerebral grey matter once the effect of prevalent cardiovascular disease and other factors are taken into account. We specifically hypothesized that: 1) participants with elevated tHcy would show a greater degree of grey matter loss compared to those with normal levels of tHcy, 2) participants with elevated tHcy would have worse performance on tests of general intellectual functioning, memory and psychomotor speed than people with normal tHcy, and that 3) these differences would be independent of the presence of prevalent cardiovascular disease and other vascular risk factors.

## Methods

### Study design

This is a cross-sectional analysis of participants aged 46–85 years who were recruited as part of the Heart-Mind study [20], which was designed to determine the impact of cardiovascular disease on cognition and brain structure over time. Data were collected between May 2006 and March 2009 in Perth, Western Australia.

### Participants

This is a convenience sample of community-dwelling adults recruited through advertisements in the local media or referrals from general practitioners and hospital-based clinicians. Referring hospitals included Royal Perth Hospital, Fremantle Hospital and Sir Charles Gairdner Hospital. The Human Research Ethics Committees of Royal Perth Hospital, Sir Charles Gairdner Hospital and South Metropolitan Area Health Service approved the study protocol and participants offered written informed consent. The research was conducted in accordance with the Declaration of Helsinki.

We excluded from participation people with: 1) prior clinical history of stroke or cardiac arrest, 2) formal contraindication to assessment with magnetic resonance imaging (MRI), 3) a score of 23 or less on the Mini-Mental State Examination (MMSE) [21], 4) history of myocardial infarction within the preceding 30 days, 5) clinically significant depressive or anxiety symptoms as determined by Hospital Anxiety and Depression Scale (HADS) sub-scores of greater than 10 [22], 6) visual or hearing impairment that was sufficiently severe to interfere with the assessments, 7) difficulty communicating in written and spoken English and 8) a serum creatinine level >0.20 mmol/L.

### Outcomes of interest

There were two main outcomes of interest: cerebral grey matter volume and cognitive function. Brain images were acquired with a 1.5 Tesla Siemens® Symphony MRI scanner (TR: 2830 ms, TE: 4.48, flip angle: 15, matrix size: 256×256×172, voxel size: 0.9 mm^3^). DICOM data were then converted to NIfTI file format using MRIcron (http://www.mricron.com). We used Statistical Parametric Mapping version 8 (SPM8 release 4010) software to process the data on Matlab® 2010b (version 7.11.0.584). T1-weighted images were skull-stripped, normalized and segmented into grey matter (GM), white matter (WM) and cerebral spinal fluid (CSF) with the VBM8 toolbox, version 359 (http://www.dbm.neuro.uni-jena.de/vbm). We subsequently created GM and WM templates using the SPM8 Dartel toolbox. Non-linear only deformations were used to warp skull-stripped images to the created templates using the VBM8 toolbox. The resultant volumetric images (voxel size: 1.5 mm^3^) were smoothed with a FWHM = 10 mm Gaussian kernel. Images were acquired within eight weeks of the clinical assessment (see below). Due to acquisition artifact on a few images, parts of the left temporal lobe were excluded from statistical analyses.

General cognitive functioning was assessed with the Cambridge Cognitive Examination of the Elderly (CAMCOG) [23]. The CAMCOG is a brief neuropsychological test that assesses a broad range of cognitive functions including memory, attention, concentration, calculation, executive functioning, language, praxis and gnosis. The CAMCOG yields a total score of 105 with sub-scores for the various cognitive domains. The CAMCOG is a well-validated instrument with population-derived normative values available [23]. Other cognitive outcomes of interest include the immediate, short and delayed recall of the California Verbal Learning Tests (CVLT) [24] and the Digit Copy and Coding subtests of the Wechsler Adult Intelligence Scale [25]. The latter two tests measure psychomotor planning and speed.

### Main exposure

We collected fasting blood samples between 08:00 and 09:00 at the Royal Perth Hospital Department of Biochemistry and these were processed immediately to extract plasma and serum, which were then batched and stored at −80°C until assayed. Total plasma homocysteine concentration was determined by reverse phase high performance liquid chromatography after treatment with tributylphosphine, deproteinization and fluorogenic derivatization using the method of Araki and Sako [26]. The coefficient of variation ranged from 3% to 7%. High tHcy was defined as a concentration of 15 µmol/L or greater [27].

### Other variables of interest

Demographic, lifestyle and clinical data were obtained at the initial assessment. Participants were asked about their age, marital status, education (duration and highest level attained), exercise pattern and smoking history. They were also asked about a history of cardiovascular diseases, including symptoms of congestive heart failure (CHF), angina and myocardial infarction, stroke, diabetes, dyslipidemia and classified according to the New York Heart Association (NYHA) rating scale. The MMSE was administered in order to exclude people who showed evidence of cognitive impairment.

Participants with cardiovascular diseases consisted of people with stable CHF or with a positive history of ischemic heart disease (IHD). People with CHF showed evidence of left ventricular systolic dysfunction (ejection fraction – EF – less than 0.4) and had a minimum six-month history of clinical symptoms consistent with CHF [28]. We excluded participants with CHF class IV of the NYHA Classification [29] due to significant morbidity and high risk of mortality [30]. The diagnosis of ischemic heart disease was based on clinical and biochemical evidence of past myocardial infarction (i.e. raised creatinine kinase MB fraction, troponin I, troponin T and ECG changes).

Alcohol use was quantified in terms of standard drinks consumed per week and participants were classified into safe and risky drinkers based on the current National Health and Medical Research Council (NHMRC) of Australia's safe drinking guidelines of no more than two standard drinks a day (http://www.nhmrc.gov.au/publications/synopses/ds10syn.htm). Participants who reported a total of 150 minutes or more of vigorous or non-vigorous activity per week were considered physically active [31]. We also measured participants, blood pressure weight and height and calculated their body mass index (BMI, kg/m^2^).

All participants underwent an echocardiographic examination to determine their left ventricular EF, which was calculated with the following formula: final diastolic volume – final systolic volume/final diastolic volume.

### Statistical analysis

Data were analyzed with Stata version 11.2 (StataCorp, Texas). Descriptive statistics were used to investigate the distribution of data according to tHcy status. We used Student t tests to compare differences between normal and high tHcy for normally distributed continuous variables and the Mann-Whitney test (z statistic) for ordinal data. Skewed continuous variables were log-transformed to assist in the analysis and values were reported as geometric means. Chi-square or Fisher's exact tests were used to compare between-group distribution of proportions. The association between cognitive outcomes and grey matter volume was explored with a Pearson's correlation (r statistic).

We used analysis of covariance (ANCOVA – F statistic) to analyze the cognitive outcomes and overall grey matter volume differences between participants with normal (tHcy<15 µmol/L) and high tHcy (tHcy≥15 µmol/L). We analyzed these outcomes in three different ways: 1) unadjusted, 2) adjusted for age only, and 3) adjusted for the presence of diabetes, age, gender, hypertension, smoking and prevalent cardiovascular disease. Participants' cardiovascular disease status was categorized into 0 (no cardiovascular disease), 1 (IHD) and 2 (CHF) and this was entered as a covariate in the adjusted model. These covariates comprised the best-fit explanatory model of cognitive function and grey matter volume according to tHcy status (all associated with a p value of <0.05), and they all had a significant independent effect on tHcy and cognitive function.

The selection of variables for the analysis of brain images followed the same principles. We used a full 2×2 factorial SPM design to model regional grey matter volume differences between the groups. There was no need to covariate for total intracranial volume during statistical analysis as nonlinear deformations were used for normalization. We declared as significant a between group difference of at least 100 voxels associated with a maximum p-value of 0.01. The analysis was not corrected for multiple comparisons.

## Results

### Population characteristics

Table 1 summarizes the characteristics of the study population according to tHcy status. Participants with elevated tHcy were on average more than eight years older (t = −5.01, p<0.001) and were more likely to be male (χ^2^ = 5.75, p = 0.016). These individuals were also more likely to be current smokers (Fishers exact test, p = 0.029), have CHF (χ^2^ = 6.01, p = 0.014), diabetes (χ^2^ = 7.62, p = 0.006) and hypertension (χ^2^ = 7.52, p = 0.006).

**Table 1:** Participant characteristics according to tHcy status.

	tHcy<15 µmol/L N = 106	tHcy≥15 µmol/L N = 49	Statistic	P value
**Demographics**				
Age, mean (SD)	65.5 (10.1)	73.7 (7.9)	t = −5.01	<0.001
Male gender, n (%)	54 (50.9)	35 (71.4)	χ^2^ = 5.75	0.016
Years of education, mean (SD)	12.5 (3.1)	11.9 (3.6)	t = 1.09	0.276
Married, n (%)	79 (74.5)	37 (75.5)	χ^2^ = 0.00	0.971
**Lifestyle**				
Ever smoked, n (%)	55 (51.9)	33 (67.3)	χ^2^ = 3.26	0.071
Current smoker, n (%)	3 (2.8)	6 (12.2)	FET	0.029
Physically active, n (%)	29 (27.4)	10 (20.4)	χ^2^ = 0.86	0.354
BMI, mean (SD)	26.5 (4.3)	26.8 (3.6)	t = −0.39	0.700
Risky alcohol use, n (%)	36 (34.0)	12 (24.5)	χ^2^ = 1.41	0.236
**Clinical**				
IHD, n (%)	39 (36.8)	17 (34.7)	χ^2^ = 0.06	0.800
CHF, n (%)	18 (17.0)	17 (34.7)	χ^2^ = 6.01	0.014
Diabetes, n (%)	13 (12.3)	15 (30.6)	χ^2^ = 7.62	0.006
Hypertension, n (%)	42 (39.6)	31 (63.3)	χ^2^ = 7.52	0.006
Dyslipidemia, n (%)	58 (54.7)	28 (57.1)	χ^2^ = 0.08	0.778
Systolic BP, mean (SD)	135.2 (19.5)	136.2 (23.6)	t = −0.27	0.787
Diastolic BP, mean (SD)	81.8 (11.4)	79.3 (12.6)	t = 1.19	0.236
Ejection fraction, mean (SD)	61.7 (15.5)	56.0 (19.1)	t = 1.98	0.049
HADSA, mean (SD)*	3.4 (2.1)	1.9 (2.0)	z = 3.85	<0.001
HADSD, mean (SD)*	2.2 (1.9)	2.2 (1.8)	z = −0.18	0.859

* Geometric mean.

Abbreviations: tHcy – total plasma homocysteine, FET – Fisher's exact test, BMI – body mass index, IHD – ischemic heart disease, CHF – congestive heart failure, BP – blood pressure, HADSA – hospital anxiety and depression scale anxiety score, HADSD - hospital anxiety and depression scale depression score.

### MRI findings

**Figure 1:**
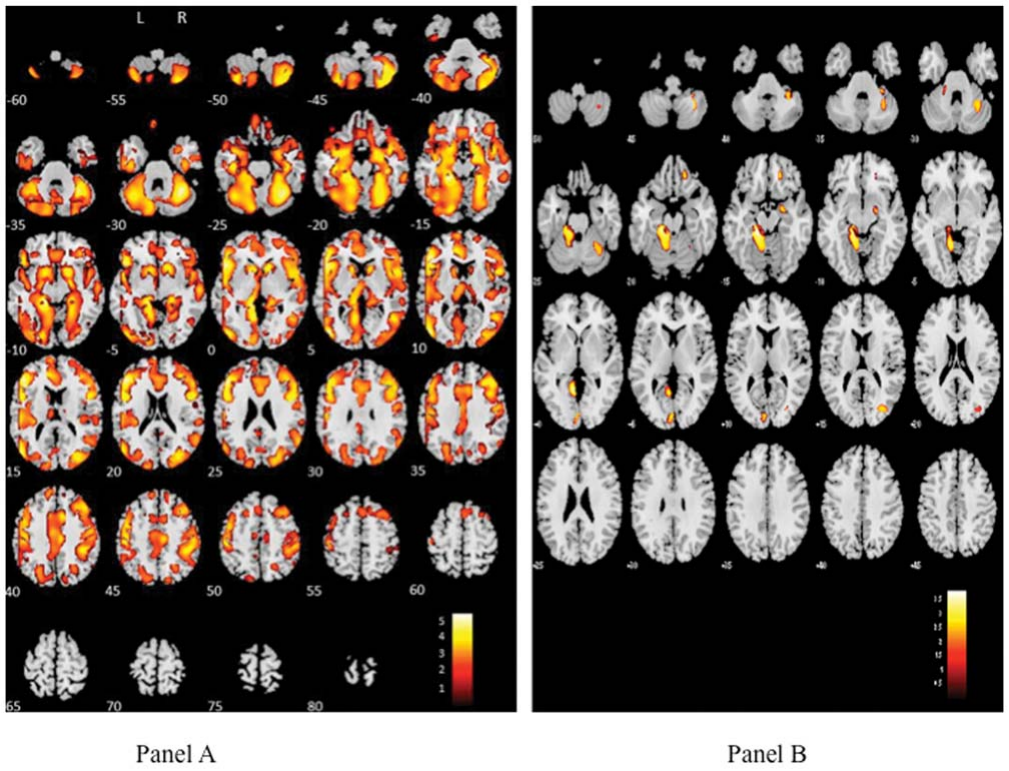
Grey matter density according to homocysteine status. This figure illustrates the regions in which participants with tHcy<15 µmol/L had higher volume of grey matter compared to participants with elevated tHcy. Panel A shows the crude analysis and panel B shows the analysis following adjustments for age, gender, diabetes, hypertension, current smoking and prevalent cardiovascular disease. The color illustrates the magnitude of the differences with white (upper portion of the color palette) being the greatest.

Participants with high tHcy (n = 49) showed evidence of widespread loss of grey matter volume compared with people with tHcy<15 µmol/L (n = 106) although differences between the groups were much less pronounced once the analysis was adjusted for age, gender, diabetes, hypertension, current smoking and prevalent cardiovascular diseases. The relevant brain regions included the left inferior occipital gyrus (Montreal Neurological Institute coordinates [MNI] in mm -48, -80, 6, t = 5.56), left parahippocampal gyrus (MNI -20, -35, -12, t = 5.48) and left middle frontal gyrus (MNI -47, 30, 11, t = 5.39) (cluster size = 138054) in the unadjusted analysis (Panel A, Figure 1). There were also significant differences in the left superior frontal gyrus (cluster size = 192, MNI -21, 18, 53, t = 2.83) and right superior (MNI 12, 45, 44, t = 2.61) and medial frontal gyrus (MNI 15, 34, 44, t = 2.49) (cluster size = 123).

The differences were much less pronounced in the multivariate model (Panel B, Figure 1). Participants with high tHcy had lower grey matter volume in the left culmen of the cerebellum (MNI -14, -33, -15, t = 3.83; -9, -54, 0, t = 3.46; -14, -30, -22, t = 3.26) (cluster size = 2898), the right cerebellar tonsil (MNI 33, -54, -45, t = 3.06; 32, -45, -42, t = 2.82) (cluster size = 876), the left occipital lingual gyrus (MNI -5, -89, 8, t = 2.88) (cluster size = 301), the middle gyrus (MNI 34, -78, 17, t = 2.79) and cuneus of the right occipital lobe (MNI 27, -77, 14, t = 2.72) (cluster size = 216), the right medial frontal gyrus (cluster size = 175, MNI 18, 39, -15, t = 3.09) and the right lentiform nucleus (cluster size = 162, MNI 23, -5, -13, t = 2.71).

### Cognitive outcomes

We examined cognitive scores and total grey matter volume of participants with high tHcy compared with normal tHcy. Total grey matter volume was derived from the SPM analyses. Once again, we adjusted the analyses for age, diabetes, hypertension, current smoking, prevalent cardiovascular disease and gender. Table 2 summarizes the results of these analyses showing that participants with high tHcy had worse cognitive function and significantly lower grey matter volume than those with normal tHcy, although these differences were no longer significant in the fully adjusted models. Cognitive tests included measures of general cognitive function, immediate recall, delayed recall and psychomotor planning and speed. The adjusted CAMCOG score of participants with high compared with normal tHcy was 0.8 points lower (95%CI −2.9 to 1.3, p = 0.476), but this difference was not statistically significant.

**Table 2:** Cognitive outcomes and total grey matter volume according to tHcy status.

	tHcy<15 µmol/L N = 106	tHcy≥15 µmol/L N = 49	F statistic	P value	Age adjusted P value	Fully adjusted P value*
CAMCOG, mean** (SD)	94.8 (1.1)	90.9 (1.1)	13.54	<0.001	0.010	0.224
CVLT immediate recall, mean (SD)	46.5 (10.8)	41.9 (11.5)	5.78	0.017	0.412	0.920
CVLT short delay recall, mean (SD)	9.5 (3.1)	7.9 (3.6)	8.08	0.005	0.352	0.997
CVLT long delay recall, mean (SD)	9.9 (3.4)	8.3 (3.4)	7.97	0.005	0.298	0.777
CVLT long delay cued recall, mean (SD)	10.5 (3.1)	9.1 (3.4)	7.00	0.009	0.331	0.791
Digit code, mean (SD)	57.8 (14.7)	44.9 (13.5)	26.46	<0.001	0.020	0.194
Digit copy, mean (SD)	94.1 (23.4)	79.7 (20.8)	13.39	<0.001	0.048	0.189
Grey matter volume, mean (SD)	561.6 (52.9)	538.0 (45.3)	7.33	0.008	0.164	0.208

* Adjusted for age, diabetes, gender, hypertension, current smoking and prevalent cardiovascular disease.

** Geometric mean.

Abbreviations: tHcy – total plasma homocysteine, CAMCOG – Cambridge cognitive examination of the elderly, CVLT – California verbal learning test.

It is possible that the regional grey matter loss observed in individuals with high tHcy may, at least in part, explain the poorer cognitive function in these individuals. In our study however, regional brain changes in grey matter (data not shown) were essentially unchanged once cognitive scores were included in the model. Total grey matter volume showed poor association with cognitive function (Pearson correlation coefficient r = 0.18).

### Subgroup analysis

Cardiovascular disease and elevated tHcy have been independently associated with cognitive impairment and brain atrophy, and with each other. Consequently, it is possible that our inclusion of participants with prevalent cardiovascular disease may have biased our findings towards a positive association. Hence, we performed a subgroup analysis limited to participants who showed no clinical or laboratory evidence of CHF or IHD (n = 64). Fifteen of those had high tHcy. The latter showed evidence of widespread grey matter volume loss compared to those with normal tHcy (n = 49) although these differences were markedly attenuated once the analyses were adjusted for age, gender, hypertension, current smoking and diabetes. The significant brain areas in the adjusted model were the left (MNI-9, -98, 2, t = 2.95) and right (MNI 9, -81, -3, t = 3.36) lingual gyri and left cuneus (MNI -6, -87, 9, t = 2.76) (cluster size = 829), the right cerebellar tonsil (cluster size = 811, MNI 33, -59, -49, t = 3.46), the culmen of the left cerebellum (cluster size = 589, MNI -9, -54, 0, t = 3.17; -5, -56, -18, t = 2.98), the declive of the left cerebellum (cluster size = 124, MNI -12, -80, -15, t = 2.94) and the left cerebral postcentral gyrus (cluster size = 119, MNI -54, -17, 33, t = 2.71).

Individuals with elevated tHcy performed worse on all cognitive measures but these differences were no longer apparent after adjustment for age, diabetes, hypertension, smoking and gender. The mean CAMCOG score in those with elevated tHcy was 90.7 (SD 1.1) compared to 95.7 (SD 1.1) with an adjusted p value of 0.276. Grey matter volume was lower in people with high tHcy but this difference was not statistically significant (532.8 versus 554.7, adjusted p = 0.187).

## Discussion

### Main findings

The results of this cross-sectional study show that adults with high tHcy have lower grey matter volume in a wide range of brain regions, although differences relative to people with normal tHcy are limited once prevalent cardiovascular diseases and other relevant confounding factors are taken into account in the analysis. Additionally, individuals with elevated tHcy perform worse on tests of global cognitive function, memory and psychomotor speed, but these differences are no longer apparent once possible confounding is taken into account.

### Limitations

This study has weaknesses and strengths that merit comment. We recruited a well-characterized group of community-dwelling participants with well-defined cardiovascular disease profile, which allowed us to adjust our analyses for their effect. We acknowledge, however, that the generalizability of our findings could be limited due to recruitment of motivated and referred volunteers. This type of recruitment bias would have led to a selection of healthier participants and minimization of differences between the groups, thereby decreasing the power of the study to declare as significant differences between the groups. Hence, the external validity of our findings is uncertain. The cross-sectional nature of our study design limits our ability to infer a causal relationship between high tHcy, cognitive function and regional brain changes, although the rationale for this study is consistent with previously reported findings, and the hypothesis linking high tHcy to neurodegeneration and cognitive decline is supported by strong biological plausibility [32], [33], [34], [35]. We also acknowledge that our participants were relatively young and free of significant cognitive impairment and this has limited our ability to investigate the effect of tHcy across the entire range of cognitive function and age.

We relied on a single estimation of tHcy and the duration of exposure or variations in the concentration of tHcy over time could not be taken into account in the analyses. Current evidence suggests that a single measurement may underestimate the risk of the outcome of interest because of regression dilution bias [36]. We used the cut-point of 15 µmol/L to define high tHcy, and although this may be seen as a relative arbitrary definition, it does take into account the results of numerous studies showing that people with tHcy above these levels experience adverse health events more frequently than their counterparts. Unmeasured factors, such as participants' vitamin B12 and folate concentration and supplementation, renal function and apolipoprotein E genotype status may have also contributed to confound the observed associations. However, as our results show that the adjusted differences in cognitive performance and grey matter volume between participants with high versus normal tHcy are minimal and of questionable clinical significance, it seems improbable that these unmeasured factors would have enhanced the differences between the groups. In fact, the opposite is more likely to be true.

We used standardized procedures and statistical software to analyze the imaging data. Voxel based morphometry is an approach susceptible to type I error when used in exploratory studies such as ours. We attempted to minimize this risk by accepting as significant only brain regions where between group differences were greater than 100 voxels and were associated with alpha of 1% or less. Automated brain volume measurements are generally accurate although measurement error of up to 5% may occur [37]. This could be related to voxel misclassification, unclear tissue edges and head tilt [38]. For this reason, we excluded from the analysis a portion of the left temporal lobe where, we suspected, tissue misclassification could have occurred. In addition, we adjusted these analyses for a number of factors that commonly affect grey matter volume, although we did not have access to information about duration of prevalent cardiovascular diseases and their treatment.

Our analyses included 91 participants who had common and well-defined cardiovascular diseases (CHF and IHD). It is conceivable that high tHcy leads to loss of cerebral grey matter and a decline in cognitive function by increasing cerebrovascular pathology, which would suggest that adjustment for prevalent cardiovascular morbidity would represent an over-adjustment of the analyses. We attempted to address this possibility by completing a series of posthoc analyses that showed that the pattern of structural and functional brain changes remained stable, although we acknowledge that this subgroup analysis was associated with loss of statistical power.

Finally, cognitive function was assessed with well-validated standardized tools, but due to the cross-sectional design we are unable to estimate the effect of tHcy on changes in cognitive function over time. Also, due to the exclusion of cognitively impaired individuals, we are unable to comment on the potential impact of tHcy on those with existing cognitive impairment or dementia.

### Interpretation of the findings

We found an inverse association between tHcy and grey matter volume in our sample but this all but disappeared once prevalent cardiovascular disease and risk factors were taken into account. This suggests that the observed effect of tHcy on brain structure is most likely mediated by age, gender and cardiovascular factors. We concede that we might have over-adjusted the analyses given the possibility that vascular factors may lie on the causal pathway between tHcy and brain structure and function, but note that tHcy is not the most important risk factor for cardiovascular disease (http://www.heartfoundation.org.au). Hence, reducing the prevalence of other risk factors for cardiovascular disease may be more important for brain structure and function than reducing tHcy. Firbank and colleagues [39] studied 80 hypertensive subjects aged 70–90 years over a two-year period. They found that tHcy levels correlated with hippocampal (p = 0.004) and white (p = 0.006), but not grey (p = 0.6) matter atrophy rates. These findings are in contrast, however, with data from the Rotterdam Scan study [40], which showed that older adults had on average 0.23 units of cortical atrophy (95%CI 0.07 to 0.38) per standard deviation increase in tHcy. Their analyses were adjusted for relevant vascular risk factors, although could not account for the presence of cardiovascular disease as in our sample.

We found a small but statistically non-significant association between high tHcy and poorer cognitive function. The differences in cognitive function between the groups could not be explained by regional grey matter differences despite the weak correlation between grey matter volume and cognition as measured by the CAMCOG. These findings are in contrast to data from the VITACOG trial [41], [42]. In that randomized trial, B-vitamins reduced brain atrophy (0.76% per year for B vitamin group versus 1.08% for controls, p = 0.001), which in turn was associated with cognitive changes over time (r = −0.36, p<0.001). Participants in this trial however were older than in our study (≥70 years) and had established cognitive impairment.

Taken together, our results suggest that the effects of high tHcy on brain structure and function seem mostly due to the presence of cardiovascular disease and increasing age. If that is the case, lowering tHcy with B-vitamins will have limited effect on neurocognitive outcomes if the concomitant impact of cardiovascular diseases is not mitigated at the same time. Indeed, efforts to improve cognitive function by lowering tHcy through B-vitamin supplementation have been mostly unsuccessful to date [15]. Additionally, homocysteine lowering trials for the secondary prevention of cardiovascular disease have also returned negative results [43]. This is consistent with the view that preserving cognitive function in later life is dependent on a number of factors and that the chances of success will be greater if we focus on risk reduction across a number of areas including lifestyle factors, disease states and vascular risk. Recent reports suggest that a 10–25% reduction in diabetes, midlife hypertension, midlife obesity and smoking together with depression, low educational attainment and cognitive inactivity could potentially prevent 1.1–3.0 million cases of Alzheimer's dementia worldwide [44], [45].

### Conclusion

Our results indicate that the association between high tHcy and cognitive function and brain structure is largely driven by its association with increasing age and cardiovascular diseases. These findings suggest that interventions focused on the reduction of tHcy alone will have limited impact on neurocognitive outcomes if they fail to reduce the prevalence of cardiovascular diseases. We suggest that effective preventive measures will need to take into account, at the same time, the numerous risk factors for cognitive decline in later life.
